# Effect of implementation of the MORE^OB^ program on adverse maternal and neonatal birth outcomes in Ontario, Canada: a retrospective cohort study

**DOI:** 10.1186/s12884-019-2296-5

**Published:** 2019-05-03

**Authors:** Deborah Weiss, Deshayne B. Fell, Ann E. Sprague, Mark C. Walker, Sandra Dunn, Jessica Reszel, Wendy E. Peterson, Doug Coyle, Monica Taljaard

**Affiliations:** 1 Better Outcomes Registry & Network (BORN) Ontario, 401 Smyth Road, Ottawa, Ontario K1H 8L1 Canada; 2 Children’s Hospital of Eastern Ontario (CHEO) Research Institute, 401 Smyth Road, Ottawa, Ontario K1H 8L1 Canada; 3 Ottawa Hospital Research Institute (OHRI), 501 Smyth Road, Ottawa, Ontario K1H 8L6 Canada; 4 University of Ottawa, 451 Smyth Road, Ottawa, Ontario K1H 8M5 Canada; 5 University of Ottawa, 600 Peter Morand Crescent, Ottawa, Ontario K1G 5Z3 Canada; 6 OHRI, 1053 Carling Avenue, Ottawa, Ontario K1Y 4E9 Canada

**Keywords:** Obstetrics, Patient safety, Outcome evaluation, Safety culture, Adverse outcomes

## Abstract

**Background:**

In 2002, the MORE^OB^ (Managing Obstetrical Risk Efficiently) obstetrical patient safety program was phased-in across hospitals in Ontario, Canada. The purpose of our study was to evaluate the effect of the MORE^OB^ program on rates of adverse maternal and neonatal outcomes.

**Methods:**

A retrospective cohort study, using province-wide administrative hospitalization data. We included maternal and neonatal records between fiscal years 2002–2003 and 2013–2014, for deliveries taking place at the 67 Ontario hospitals where the MORE^OB^ program was implemented between 2002 and 2012. After accounting for institutional mergers and excluding very small hospitals, 55 hospitals (1,447,073 deliveries) were included.

Multivariable logistic and linear mixed effects regression analysis were used, accounting for secular trends, within hospital correlation and over time correlation, and adjusting for a maternal comorbidity index, hospital annual birth volume, and level of care.

The main outcome measure was a composite individual-level indicator of incidence of any adverse events, and a hospital-level score, called the Weighted Adverse Outcome Score (WAOS) capturing both maternal and neonatal adverse outcomes.

**Results:**

Across the 12 years of follow up, there were 98,789 adverse maternal and neonatal outcomes, a rate of 6.83 per 100 deliveries (6.66 per 100 occurring before, 6.91 per 100 during, and 6.84 per 100 after program implementation). The multivariable analysis found no statistically significant decrease in adverse events associated with program implementation (OR for adverse events after versus before =1.11 (95% CI: 1.06 to 1.17, change in mean WAOS score after minus before =0.15 (− 0.36 to 0.67)).

**Conclusions:**

We did not find a reduction in the incidence of maternal and neonatal adverse outcomes associated with the MORE^OB^ program, and small yet statistically significant increases in some adverse events were observed.

**Electronic supplementary material:**

The online version of this article (10.1186/s12884-019-2296-5) contains supplementary material, which is available to authorized users.

## Background

In high-resource settings such as Canada, maternal and neonatal adverse events during labour and birth are relatively rare [1, 2]. Yet, when poor outcomes occur they have the potential to cause serious long term consequences for both mother and infant, and to be extremely costly for families and insurers [3–5]. For this reason, a number of obstetrical patient safety programs have been designed, with the goal of reducing adverse maternal and neonatal events [4–7].

In 2001, the Society of Obstetricians and Gynaecologists of Canada developed a program to improve patient safety. The Managing Obstetrical Risk Efficiently (MORE^OB^) program was initially pilot-tested in the Canadian province of Ontario in 2002, and subsequently expanded across Canada and elsewhere [8]. In 2007, the Society of Obstetricians and Gynaecologists of Canada partnered with the Healthcare Reciprocal of Canada to form Salus Global, the organization that is now responsible for the administration of the MORE^OB^ program [8].

MORE^OB^ is a patient safety, professional development and performance improvement program for caregivers and administrators in hospital obstetric units. It aims to improve patient safety and reduce adverse events, improve communication and teamwork and foster evidence-based obstetrical care [9]. The program is offered in three modules over a three-year period [8]. It has been reported that participation in the MORE^OB^ program results in positive changes in workplace environment and safety culture [8], and increases the clinical knowledge of participants [8]. A recently published study using health insurer data from Ontario, Canada found decreases in frequency and costs associated with reportable events for the health insurer [10]. In addition, an evaluation of clinical outcomes in the Canadian province of Alberta reported a statistically significant reduction in 3rd and 4th degree perineal lacerations following implementation of Module 1 of the program, which was sustained across all modules, as well as a significant reduction in severe neonatal morbidity following Modules 2 and 3 [11]. There were no statistically significant differences in rates of other clinical outcomes such as fetal mortality, maternal infection or postpartum hemorrhage; however, the study was likely not sufficiently powered to detect changes in these infrequent outcomes [11]. While there were numerous strengths of the Alberta evaluation, there were also several limitations, including the use of a simple before-and-after study design and the short follow-up period which limited inference on program impact over the longer term.

Between 2002 and 2012, 67 Ontario hospitals adopted the MORE^OB^ program, and as of 2014, 63 of these had completed the three modules. As part of a larger mixed-methods evaluation of the MORE^OB^ program, we report here the results of an analysis assessing the effect of implementation of the MORE^OB^ program on rates of adverse maternal and neonatal outcomes in the province of Ontario using a retrospective cohort study.

## Methods

### Study design

**Fig. 1:**
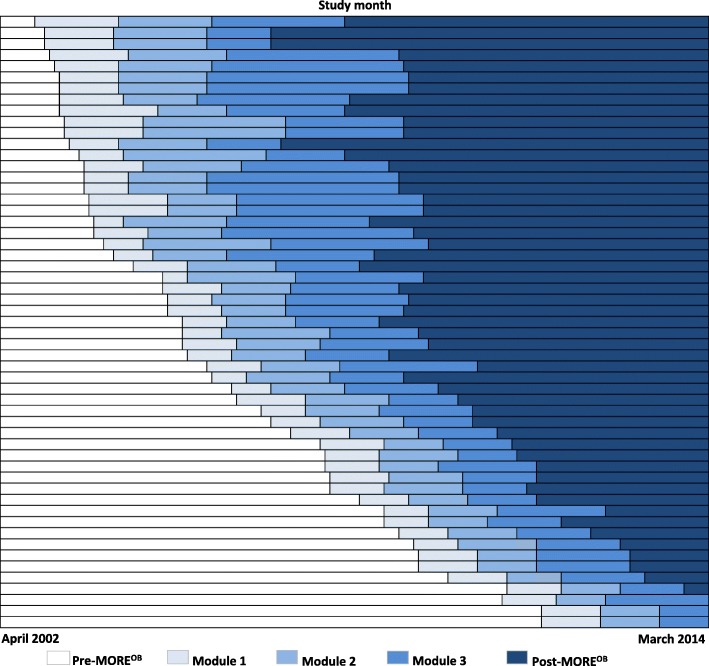
Program implementation timing by month, at 55 hospitals, Ontario, Canada, 2002 to 2014

MORE^OB^ program implementation over the 12-year study period, for each included hospital, is presented in Fig. 1. Designing a robust evaluation of the MORE^OB^ program implementation was challenging for several reasons, including the staggered implementation times across the hospitals and the long and variable program implementation phases [12–14]. We approached our analysis using a quasi-experimental retrospective cohort design with analysis based on the approach used for stepped wedge trials. The stepped wedge is a type of study design which can be used to evaluate interventions implemented in phases at different points over time [15, 16]. In stepped wedge studies, outcomes are assessed repeatedly before and after implementation, and the overall effectiveness of the intervention is determined by comparing data points in the post-intervention section of the wedge, to those in the pre-intervention section.

### Study population

Initially, the 67 hospitals that implemented the MORE^OB^ program prior to 2012 were included. The study population was comprised of the mother-newborn dyads born at these hospitals between fiscal years 2002–2003 and 2013–2014.

In preparing the dataset for analysis, we accounted for hospital mergers and closures over the course of the 12 years of follow up, which decreased the number of institutions from 67 to 63. To avoid instability in the multivariable analysis, we subsequently excluded the 8 institutions with annual birth volumes less than 250. Thus, 55 institutions were included in our final analysis.

### Data sources

We obtained a 12-year dataset of maternal-newborn hospitalization records from the Canadian Institute for Health Information’s (CIHI) Discharge Abstract Database (DAD) for Ontario hospitals between April 1, 2002 and March 31, 2014 (i.e., fiscal years 2002–2003 to 2013–2014, inclusive). The DAD includes hospital separation abstracts submitted by all acute care hospitals in Ontario. Each hospital abstract contains demographic information, medical diagnoses including most responsible diagnosis and 24 secondary diagnoses, interventions received, length of hospital stay, vital disposition at time of discharge and other data elements. Medical diagnoses are coded using the Canadian implementation of the International Classification of Diseases, 10th Revision (ICD-10-CA) from 2002 to 2003 to the present. The DAD captures over 98% of all births, including all live birth and stillbirth events occurring in Ontario hospitals through the maternal record. In addition, there are separate records for live born neonates and stillbirths that capture relevant morbidities, diagnoses and medical procedures. We linked maternal and newborn abstracts together via an encrypted maternal-newborn chart number [17].

### Primary outcomes

We analyzed two previously-developed measures of obstetric patient quality of care: a composite individual-level indicator of incidence of any adverse maternal or neonatal events, and a hospital-level score, called the Weighted Adverse Outcome Score (WAOS). Both measures capture maternal and neonatal outcomes, and were developed through repeated consultation with leaders in nursing, obstetrics and anesthesia, among others [18]. The decision to use these two composite indices was made for several reasons. Using previously-developed measures facilitates comparison between our study and previously published research [5, 6, 18–20]. Further, the MORE^OB^ program is comprehensive and multifaceted, and could affect a number of maternal and newborn outcomes. Therefore, using composite scores would better capture the potential impact of the program. Note that several modifications were required, most importantly the omission of the Apgar score, as it was not available in the hospitalization database.

The composite individual-level indicator of adverse event occurrence was based on the Adverse Outcome Index (AOI), a composite score designed to capture the quality of obstetrical care defined as “the number of deliveries complicated by one or more of the identified outcomes divided by the total number of deliveries” [18]. We modified the AOI for our analysis (henceforth, mAOI), and used it as a binary individual-level indicator, rather than as a proportion calculated at the monthly and hospital level, where a ‘1’ indicates the presence of any component of the AOI and a ‘0’ indicates no components had occurred for that individual. We decided to use an individual-level analysis for the mAOI to allow for unequal weighting based on hospital birth volume. The mAOI captures the number of deliveries affected by one or more of the adverse events included in the indicator (see Table 2 and Additional file 1). The WAOS additionally accounts for the severity of these events, by assigning weighted scores to each event (Table 2). Further, because the weights are summed, both the severity and number of events contribute to the score. For example, maternal death is assigned the highest score at 750 points per event, and 3rd or 4th degree perineal tears are assigned the lowest, at 5 points per event. If a delivery is complicated by multiple events, their scores are summed [18]. The weighted sums are then divided by the number of deliveries that took place during a particular time period (e.g., per month).

### Dataset preparation

The timing of implementation for each hospital was divided into segments based on program implementation: the pre-implementation period, the implementation period which included implementation time for each of the three MORE^OB^ modules, and the post-implementation period. Two different datasets were prepared, one for each outcome. The mAOI was defined as described above and analyzed at the individual level, and the WAOS was defined at the level of the hospital, as the sum of all the weighted composite scores, divided by the number of deliveries that occurred in that hospital. Therefore, the analysis of the WAOS required the creation of a dataset aggregated by month and hospital.

The components of the composite indices were coded from the ICD-10-CA and CCI (Canadian Classification of Health Interventions) codes in the DAD, after reviewing published lists of codes used for maternal-newborn research using the DAD [19, 21–23]. The codes used for our study, along with additional information about the components, can be found in Additional file 1. Components of the mAOI were modified based on the information that was available in the DAD dataset. Specifically, the Apgar score was unavailable and, therefore, not included, and the data quality of 3rd degree perineal tears was found to be problematic and therefore our component was restricted to 4th degree perineal tears. Information on the timing of module implementation for each hospital was obtained from Salus Global.

### Statistical analyses

#### Descriptive analyses

We used descriptive summary statistics to examine the distribution of study variables at the hospital level. Monthly time series plots of crude rates of study outcomes (mAOI, WAOS and component outcomes) over the 12-year study period were graphed by actual calendar time (see Additional file 2).

### Multivariable regression analyses

The mAOI was specified as the dependent variable and analyzed at the individual woman-level using random effects logistic regression, estimated using restricted pseudo-likelihood estimation. MORE^OB^ program implementation, coded as a step function taking the value 0 before implementation, increments of 0.25 with each subsequent module, and 1 after completion of all modules, was included as a fixed effect. To account for the underlying secular trend, (i.e., natural changes that occur over time [24]), time was modeled as a restricted cubic spline with five knots. Level of care, annual birth volume, and an obstetric comorbidity index [25] were also included as fixed effects. The previously developed and validated obstetric comorbidity index [25, 26] has been found to predict severe maternal morbidity and mortality and includes more than 20 maternal comorbidities and conditions that increase the risk of adverse pregnancy outcomes [25, 26]. To allow individual hospitals to deviate randomly from the secular trend, random intercepts and coefficients for time were specified for each hospital. The regression coefficient for MORE^OB^ program implementation was exponentiated to yield an adjusted odds ratio (aOR) for the effect of program implementation. This odds ratio compares the odds of an adverse event for a woman in a hospital after program implementation, to that of a woman in the same hospital before implementation. Three important assumptions of our model are that, after adjusting for covariates, there are no systematic differences in pre-implementation levels and trends across hospitals; that the effect of the intervention is constant over time (i.e., the models yield an estimate of the time-averaged hospital-specific effect of the intervention); and there is a gradient effect of the implementation across all modules, meaning a constant increase from the first to last modules.

The WAOS was analyzed at the hospital-level using mixed effects linear regression, estimated using restricted maximum likelihood estimation. Fixed and random effects were as described for the analyses of the mAOI, with the exception that the comorbidity index was aggregated to the level of the hospital in each month. The regression coefficient for the MORE^OB^ program implementation variable yields an estimate of the difference between the mean score post-implementation and the mean score pre-implementation.

For both outcomes, the fitted secular trend was obtained from the model and displayed graphically with the covariates (hospital level and annual birth volume, and comorbidity index) set to their mean values in the study sample.

Secondary analyses included separate models for individual components of the composite score, and examining sensitivity due to misspecification of the secular trend by specifying time as a categorical (rather than continuous) variable. Also, between-hospital heterogeneity in the effect of the program was allowed for, by adding a random effect for program implementation defined at the level of the hospital. All analyses were carried out using SAS v. 9.4.

## Results

The hospital sample was comprised of 12 level 1, 37 level 2 and 6 level 3 hospital sites, with 16 sites having annual birth volumes less than 1000, 32 sites having annual birth volumes between 1001 and 4000, and 7 sites with annual birth volumes greater than 4000 (see Additional file 3). A description of the study sample is presented in Table 1. The sample was restricted to women with singleton pregnancies. Overall, the study sample included 1,447,073 deliveries at the 55 hospitals, with 328,864 (22.7%) taking place before MORE^OB^ was implemented, 556,550 (38.5%) taking place during implementation, and 561,659 (38.8%) taking place after.

**Table 1:** Study sample characteristics of deliveries occurring in 55 institutions in Ontario, Canada, *n* = 1,447,073

*Variable*	
Hospital birth volume, n (%)	
251–500	22,547 (1.6)
501–1000	89,516 (6.2)
1001–2499	407,622 (28.2)
2500–4000	509,269 (35.2)
> 4000	418,119 (28.9)
Hospital level of care, n (%)	
1	146,205 (10.1)
2	1,023,874 (70.8)
3	276,994 (19.1)
Fiscal year of delivery, n (%)	
2002–2005	352,778 (24.4)
2005–2008	367,349 (25.3)
2008–2011	366,724 (25.3)
2011–2014	360,222 (24.9)
Timing of MORE^OB^ participation at time of delivery, n (%)	
Before start of MORE^OB^ program	328,864 (22.7)
During Module 1	114,175 (7.9)
During Module 2	176,581 (12.2)
During Module 3	265,794 (18.4)
Post-MORE^OB^	561,659 (38.8)
Duration of hospital participation in each module in months, mean (SD), Q1 to Q3	
Module 1	11.3 (3.2), 9 to 12
Module 2	17.2 (4.8), 14 to 19
Module 3	25.0 (9.0), 16 to 33
Total	53.5 (10.9), 44 to 64

*MORE*^*OB*^ Managing Obstetrical Risk Efficiently

It took hospitals on average 11.3 months to complete the first module, 17.2 months to complete the second and 25 months to complete the third. As indicated by the standard deviations and interquartile ranges, there was in fact a great deal of variability between hospital sites with respect to amount of time taken to complete the program, and program completion took longer than the 3 years that has been reported [9].

Table 2 presents the occurrence of adverse events across the 12 years of follow up. There were 98,789 adverse events, for a rate of 6.83 per hundred deliveries, with 21,898 (rate of 6.66%) events occurring before MORE^OB^ implementation, 38,462 (rate of 6.91%) occurring during implementation, and 38,429 (rate of 6.84%) occurring after. The most frequent adverse outcome was neonatal intensive care unit (NICU) admission for at least 2 days for infants with a birth weight of 2500 g or more, which had a rate of 4.22% (*n* = 61,079). The least frequent event was maternal death, with a rate of 0.005% (*n* = 69).

**Table 2:** Components and total scores for the Adverse Outcome Index and Weighted Adverse Outcome Score

*Variable*	Full Sample *n* = 1,447,073	WAOS Weights	Total WAOS points^a^	Pre-MORE^OB^ *n* = 328,864	During MORE^OB^ *n* = 556,550	Post-MORE^OB^ *n* = 561,659
	*n(%)*			*n(%)*	*n(%)*	*n(%)*
Maternal death	69 (0.00)	750	51,750	14 (0.00)	30 (0.01)	25 (0.00)
Uterine rupture	516 (0.04)	100	51,600	106 (0.03)	192 (0.03)	218 (0.04)
Maternal admission to ICU	3566 (0.25)	65	231,790	714 (0.22)	1406 (0.25)	1446 (0.26)
Unanticipated operative procedure	11,081 (0.77)	40	443,240	2112 (0.64)	4168 (0.75)	4801 (0.85)
Blood transfusion	7880 (0.54)	20	157,600	2069 (0.63)	3121 (0.56)	2690 (0.48)
4th degree tear^b^	5187 (0.36)	5	25,935	1169 (0.36)	2128 (0.38)	1890 (0.34)
Neonatal death	12,074 (0.83)	400	4,829,600	2613 (0.79)	4731 (0.85)	4730 (0.84)
Birth trauma	4045 (0.28)	60	242,700	1242 (0.38)	1641 (0.29)	1162 (0.21)
NICU admission^c^	61,079 (4.22)	35	2,137,765	13,199 (4.01)	23,584 (4.24)	24,296 (4.33)
Adverse Outcome Index^d^	98,789 (6.83)	–	–	21,898 (6.66)	38,462 (6.91)	38,429 (6.84)
	*Mean (SD)*			*Mean (SD)*	*Mean (SD)*	*Mean (SD)*
Weighted Adverse Outcome Score	4.86 (4.3)	–	–	4.52 (4.50)	5.05 (4.15)	4.97 (4.27)
Maternal age	28.86 (4.21)	–	–	27.43 (6.17)	29.06 (3.70)	29.83 (1.56)
Maternal comorbidity index	0.48 (0.18)	–	–	0.41 (0.20)	0.49 (0.17)	0.53 (0.17)
Gestational age of infant at birth in weeks	38.8 (2.0)	–	–	38.9 (2.0)	38.8 (2.1)	38.8 (2.1)

*ICU* Intensive Care Unit, *MORE*^*OB*^ Managing Obstetrical Risk Efficiently, *NICU* Neonatal Intensive Care Unit

^a^Number of events multiplied by the WAOS weight

^b^3rd degree tear data were of poor quality and therefore only 4th degree tears were included in the composite index

^c^NICU admission, for at least 2 days, or transfer within 24 h of birth to a facility with a NICU, for an infant with birth weight at least 2500 g

^d^5 min Apgar not included

Table 3 presents the results of the multivariable regression analyses of the association between MORE^OB^ implementation and adverse maternal and neonatal events when time was modelled using a restricted cubic spline. This model revealed no evidence of improvements in adverse events after MORE^OB^ implementation. In fact, for the mAOI, there was a small increase in the odds of an adverse event in the post-MORE^OB^ time period compared with the pre-MORE^OB^ time period (aOR = 1.11 95% CI: 1.06 to 1.17).

**Table 3:** Effect of implementation of MORE^OB^ on study outcomes^a^, Ontario, 2002–2014

Outcome	Primary analysis: Does not account for between-hospital heterogeneity	Secondary analysis: Accounts for between-hospital heterogeneity
WAOS, mean change (95% CI)	0.15 (−0.36 to 0.67)	0.16 (−0.41 to 0.74)
mAOI, OR (95% CI)	1.11 (1.06 to 1.17)**	1.09 (1.04 to 1.15)**
*Maternal components*		
Maternal ICU admission	0.97 (0.75 to 1.25)	0.97 (0.75 to 1.25)
Unanticipated operative procedure	1.21 (1.05 to 1.39)**	1.26 (1.06 to 1.50)**
Blood transfusion	0.83 (0.69 to 1.00)	0.83 (0.69 to 1.00)
4th degree tear	1.36 (1.11 to 1.65)**	1.36 (1.11 to 1.65)**
*Neonatal components*		
Neonatal death	0.97 (0.84 to 1.11)	0.84 (0.71 to 1.00)
Birth trauma	0.91 (0.75 to 1.10)	0.90 (0.75 to 1.10)
NICU admission	1.24 (1.17 to 1.32)**	1.13 (1.06 to 1.21)**

*AOI* Adverse Outcome Index, *ICU* Intensive Care Unit, *MORE*^*OB*^ Managing Obstetrical Risk Efficiently, *NICU* Neonatal Intensive Care Unit, *WAOS* Weighted Adverse Outcome Score

^a^All analyses adjusted for calendar time, hospital annual birth volume, hospital level of care, and maternal comorbidity index; calendar time was modelled using a restricted cubic spline function with five knots

**p < 0.05

In the analyses of individual components of the composite scores (Table 3), the MORE^OB^ program was associated with increases in maternal unanticipated operative procedures (aOR = 1.21, 95% CI: 1.05 to 1.39) and 4th degree tear (aOR = 1.36, 95% CI: 1.11 to 1.65), and NICU admission (aOR = 1.24, 95% CI: 1.17 to 1.32). While not statistically significant at the conventional 5% level, the results suggest an association with decreased maternal blood transfusions (aOR = 0.83 (95% CI: 0.69 to 1.00)) and neonatal birth trauma (OR = 0.91 (95% CI: 0.75 to 1.10)). Maternal death and uterine rupture were not analyzed separately due to low frequency of events.

The modelled secular trends for the mAOI and WAOS are presented graphically in Additional file 4. These plots display how the mAOI and WAOS vary over time, even in the absence of the MORE^OB^ program. There is evidence that both outcomes are decreasing until the start of fiscal year 2008, at which time the rates level off or slightly increase. These trends support our choice to model time using a restricted cubic spline, which allows for more flexibility in accounting for the effects of secular trends on our outcomes. Results of the secondary analyses with time modeled categorically are presented in Additional file 5.

## Discussion

### Principal findings

In our analysis of adverse maternal and neonatal outcomes in 55 Ontario hospitals enrolled in the MORE^OB^ program prior to 2012, we found no evidence of improvements in either of the composite indices after MORE^OB^ implementation. In fact, there was a small increase in the occurrence of adverse events as captured by the mAOI. In subanalyses with individual components of the composite indices, we found small but statistically significant increased odds of maternal unanticipated operative procedures, 4th degree tears, and NICU admission; on the other hand, we found evidence of non-statistically significant decreases in neonatal birth trauma and maternal blood transfusion.

### Comparison with previous studies

Two previous studies have assessed the effect of the implementation of the MORE^OB^ program. In the first study, implementation of the MORE^OB^ program in Alberta hospitals was associated with a reduction in 3rd and 4th degree perineal tears and decreases in maternal length of stay and neonatal morbidity [11]. However there were limitations to this study, including the analysis which was vulnerable to confounding by the underlying secular trend, as well as the short follow-up time period. In the Alberta study only 2.8% of deliveries occurred post-Module 3, whereas in our study 39% of deliveries occurred post-Module 3. Using a multiple baseline interrupted time series (ITS) design, the second study assessed the effect of MORE^OB^ implementation on mandatory reportable events collected by a Canadian healthcare liability insurer [10]. In that study, the authors reported a decrease of 4 mandatory reportable events at 3 years after MORE^OB^ implementation (95% CI:-0.5 to 8.1) and a decrease of 8 mandatory reportable events at 6 years post-implementation (95% CI: 1.4 to 15.1). However, this study included a smaller number of Ontario hospitals, 34, whereas the current study included 55 Ontario hospitals.

Other obstetric patient safety programs have been evaluated with mixed results. A recently published rapid review included 10 randomized controlled trials (RCT) and reported that “provider education and other quality improvement strategy combinations targeting healthcare providers may improve the safety of women and their newborns during childbirth” [7]. In an evaluation of a program implemented at an American hospital from 2004 to 2006, it was reported that the program was associated with a decrease in the AOI [5], as well as a decrease in the number of liability claims and associated payments [4]. The nine elements of the program included items such as the development of new protocols and guidelines, the hiring of an Obstetric Safety Nurse, the implementation of an Obstetric Patient Safety Committee, the involvement of Obstetric Hospitalists to provide 24-h, 7-day per week in-house coverage, as well as team training and an electronic fetal heart rate certification program [4, 5]. However, this program was implemented at one hospital, and a simple pre/post linear regression analysis was used, making it difficult to draw conclusions about what the wider impact of such a program would be. An RCT carried out at 15 American hospitals assessing the effect of a teamwork training program on the AOI reported no effect of the program on this outcome [6]. Based on the literature to date, it is difficult to conclude what elements of an obstetric patient safety program might be most likely to improve effectiveness.

### Strengths and weaknesses

In our analytical approach, we allowed for a gradient effect of MORE^OB^ implementation (from the first to the final module) but thereafter, the effect of the MORE^OB^ program was assumed to be stable. An alternative approach may have allowed for either a gradual decay or further gradual improvements after completion of all modules, using, for example, an ITS approach with segmented regression analysis allowing for both a step change and a slope change. However, the staggered implementation of the MORE^OB^ program, combined with the lengthy and variable amount of time required for hospitals to complete the program, precluded the use of an ITS approach. In a classical ITS analysis, the estimated pre-implementation trend (secular trend) is used to predict what the outcome event rate would have been at the end of the study, had the intervention not been implemented (the counterfactual estimate) [12–14]. With a long separation, using the secular trend to predict a counterfactual estimate is risky due to projection far outside the observed range of the data.

While the staggered implementation of the MORE^OB^ program, which occurred over an 8 year period, presented analytic challenges, it also provided an advantage, in that our results are less likely to be confounded by concurrent interventions or local policy changes. Using our multivariable model, we were essentially able to compare outcomes from all hospitals after implementation of the program to their own pre-implementation outcomes as well as to the outcomes from other hospitals who had not yet implemented the program at the same calendar time. As well, the use of previously developed and used [5, 6, 18–20] indicators for adverse maternal and neonatal outcomes, the mAOI and WAOS, further strengthens our results. Given the multifaceted nature of the MORE^OB^ program, we considered that indicators capturing multiple components of maternal/newborn care would be more appropriate. Further, aggregation of the components helps to address the rarity of these adverse outcomes in the Canadian healthcare setting. And, given that it had been used for research carried out in Canada [19], this allows for the comparison between our estimates and what has been reported elsewhere. The AOI rate reported by Hutcheon et al. of 5.7% is lower than the 6.8% that we report here, and the WAOS we report is also higher (4.9 versus 1.9). It is not entirely clear why these rates would differ inter-provincially across Canada, and given the severity of these outcomes, this might highlight an area for further research.

We believe using composite indices as the primary outcomes was the most appropriate choice however it is possible that this masks the effect of the program on specific maternal-newborn outcomes that were not assessed by our study. As well, our analysis was limited to the data elements available in the DAD hospitalization database, and therefore we had to use a modified version of the AOI, which did not include elements such as Apgar score or 3rd degree perineal tears. Data quality is carefully managed and documented by CIHI [27], and we made every attempt to identify any outstanding data quality issues including examining the study outcomes over time and by hospital site, and liaising with CIHI when issues were identified. Yet, it is possible that unidentified changes to coding or data quality remain. Also, the occurrence of maternal and neonatal adverse outcomes is affected by a host of factors unrelated to participation in a program such as the MORE^OB^, such as maternal age, parity and comorbidity. We attempted to account for this by including hospital level of care, birth volume and a maternal comorbidity index in our models however, it is possible that unmeasured confounding remains. An additional limitation was the lack of clinical performance measures in this evaluation, such as compliance with guidelines, safety reports, or documentation of near misses. Further, changes to workplace culture were not assessed. It has previously been reported that participation in MORE^OB^ improved workplace culture and increased knowledge [8], and it is difficult to understand how improvements to workplace culture would not result in improvements to patient outcomes. Inclusion of objective measures of behavior such as those described above, along with assessments of workplace culture might help to shed light on this issue in future studies.

### Possible explanations and implications for policymakers

It is perhaps not surprising that we were unable to detect an impact of the MORE^OB^ program on a composite indicator of adverse maternal and neonatal outcomes. In this evaluation, which is one component of a larger mixed-methods evaluation of the MORE^OB^ program, we only measured adverse outcomes. Although these are important outcomes from the perspective of patients, hospitals and policymakers, they cannot fully capture other aspects of MORE^OB^ training related to improved communication, improvements in safety culture and increases in knowledge. Our mixed-methods evaluation of the MORE^OB^ program also assessed individual participant responses, including care providers’ views about the program, changes in their levels of knowledge, and their assessments of the culture change within their organizations. Our findings from that study suggest that the MORE^OB^ program had a positive impact on these factors. It is well-established that clinical behavior and practice change takes time to become entrenched [28, 29]. As well, when a hospital embarks on the 3-year commitment to implement the MORE^OB^ program, they must also find strategies to sustain the activities and practice changes once their dedicated funding for the program runs out. If the activities and practice changes are not firmly established and culture change is temporary, there may be no detectable net benefit of the program on clinical adverse outcomes over time. The MORE^OB^ program does attempt to mitigate this effect, in that it is not just an educational program, rather every attempt is made to embed the program within the culture of each institution. A dedicated core team in each hospital carries out ongoing activities related to the program [9].

### Unanswered questions and future research

Improving patient safety and reducing adverse maternal and neonatal outcomes remain issues of critical importance in obstetrics, and our study demonstrates some of the important challenges associated with this area of research. Our study also demonstrates the critical nature of large datasets and long follow-up, which is especially important when studies are carried out in high-resource settings where adverse outcomes are rare. Great care is also needed in the selection of appropriate research and analytical methods. Future studies should look at the effect of obstetrical safety programs on other adverse outcomes such as near misses, as well as including objective measures of behavior change.

## Conclusions

We did not find improvements in a composite indicator of adverse maternal and neonatal outcomes associated with implementation of the MORE^OB^ program. Future evaluation studies should consider including other outcomes which might better capture the effect of an obstetric patient safety program.

## Additional files

Additional file 1: ICD-10-CA and CCI codes that were used to define the elements of the AOI, and additional definitions. (DOCX 16 kb)

Additional file 2: Crude outcome rates over time, Ontario, Canada, 2002 to 2014. This file includes 10 figures showing outcome rates over time for: WAOS, AOI, maternal death rate, neonatal death rate, maternal ICU admission, birth trauma, maternal unanticipated operative procedures, NICU admission, blood transfusion, 4th degree perineal tear. (PDF 514 kb)

Additional file 3: Characteristics of the hospitals included in the analysis, and the characteristics for the full sample of hospitals enrolled in the MORE^OB^ program. (DOCX 14 kb)

Additional file 4: Underlying secular trend for WAOS and mAOI, using time modeled as a continuous with a restricted cubic spline function. (PDF 214 kb)

Additional file 5: Results of sensitivity analyses for association between MORE^OB^ implementation and two primary study outcomes. This table shows the Results of sensitivity for association between MORE^OB^ implementation and two primary study outcomes. (DOCX 14 kb)

## Abbreviations

AOI: Adverse Outcome Index
aOR: Adjusted odds ratio
BORN: Better Outcomes Registry & Network Ontario
CCI: Canadian Classification of Health Interventions
CI: Confidence interval
CIHI: Canadian Institute for Health Information
DAD: Discharge Abstract Database
ICD-10-CA: International Classification of Diseases, 10th Revision
ICU: Intensive Care Unit
ITS: Interrupted Time Series
mAOI: Modified Adverse Outcome Index
MORE^OB^: Managing Obstetrical Risk Efficiently
NICU: Neonatal intensive care unit
RCT: Randomized controlled trial
SD: Standard deviation
WAOS: Weighted Adverse Outcome Score
