# Supplementary material for: Effect of implementation of the MOREOB program on adverse maternal and neonatal birth outcomes in Ontario, Canada: a retrospective cohort study
Source: BMC Pregnancy Childbirth. 2019 May 3;19:151. doi: 10.1186/s12884-019-2296-5 (PMC6500060; doi:10.1186/s12884-019-2296-5)
Supplement: Supplementary file 1 — ICD-10-CA and CCI codes that were used to define the elements of the AOI, and additional definitions. (DOCX 16 kb) [file 12884_2019_2296_MOESM1_ESM.docx]

**Additional file 1: ICD-10-CA and CCI codes that were used to define the elements of the AOI, and additional definitions**

| **From original AOI**[11] | **ICD/CCI codes** | **Additional definitions** |
| --- | --- | --- |
| Maternal death | O95, O97 |  |
| Intrapartum or neonatal death > 2,500g | b_rectype=1 (stillbirth)  baby_discharge_disposition=07 and baby age less than 28 days |  |
| Uterine rupture | O71.18 |  |
| Maternal admission to ICU | 1.GJ.50.CA-NG  1.GJ.50.CA-TS  1.GZ.31.CB-ND  1.GZ.31.CA-ND  1.GZ.31.CR-ND  1.GZ.31.GP-ND  1.GZ.38.JA-ND  1.GZ.38.JA-NE  1.GZ.30.CJ  1.GZ.30.JH  Transfer to field (SCU fields) | Includes proxy measures; intubation, receipt of positive pressure ventilation, or resuscitation as well as the fields indicating transfer to a Special Care Unit |
| Neonatal birth trauma, ≥ 2,000g | P10.1  P10.2  P10.3  P12.2  P13 excluding P13.4  P11.5  P11.3  P14.0, P14.1, P14.3  P11.4, P14.2, P14.8, P14.9 | Includes cerebral hemorrhage due to birth injury, skeletal injuries due to birth, injuries to spine and spinal cord, brachial plexus, facial nerve or other nerves. |
| Unanticipated maternal operative procedures | 5.MD.60.KE  5.MD.60.RC 5.MD.60.CB  5.MD.60.RD  1.RM.89.LA (exclude if 1PL74, 1RS74 or 1RS80 also present)  1RM.87.LA-GZ  1.PZ.21^^  5.PC.73.JS  5.PC.73.JT  5.PC.80.JR  5.PC.80.JM  1.NK.80^^  1.NM.80^^  (1.RM.13^^ or 1.KT.51 or 5.PC.91.LA) + O72  5.PC.91.GC  5.PC.91.GA  5.PC.91.HT | Includes procedures to control post-partum bleeding such as D and C following delivery, control of post-partum hemorrhage by ligation or embolization, hysterectomy, etc. |
| Admission to NICU > 2,500g and for > 24hours | SCU number = 50, 51, 52, 53 (NICU Unit)  Transfer to SCU unit  Date of transfer within 1 day of birth  Total LOS in SCU ≥ 2 days |  |
| APGAR < 7 at 5 minutes | Not available |  |
| Blood transfusion | 1LZ19HHU1 1LZ19HHU9 1LZ19HMU1 1LZ19HMU9  Z51.3  CIHI Blood Transfusion Field |  |
| 4^th^ degree tear | O70.3 | 3^rd^ degree tear data were problematic therefore these were not included. |
