# Supplementary material for: Effect of implementation of the MOREOB program on adverse maternal and neonatal birth outcomes in Ontario, Canada: a retrospective cohort study
Source: BMC Pregnancy Childbirth. 2019 May 3;19:151. doi: 10.1186/s12884-019-2296-5 (PMC6500060; doi:10.1186/s12884-019-2296-5)
Supplement: Supplementary file 2 — Crude outcome rates over time, Ontario, Canada, 2002 to 2014. This file includes 10 figures showing outcome rates over time for: WAOS, AOI, maternal death rate, neonatal death rate, maternal ICU admission, birth trauma, maternal unanticipated operative procedures, NICU admission, blood transfusion, 4th degree perineal tear. (PDF 514 kb) [file 12884_2019_2296_MOESM2_ESM.pdf]

Additional file 3: Crude outcome rates over time, Ontario, Canada, 2002 to 2014

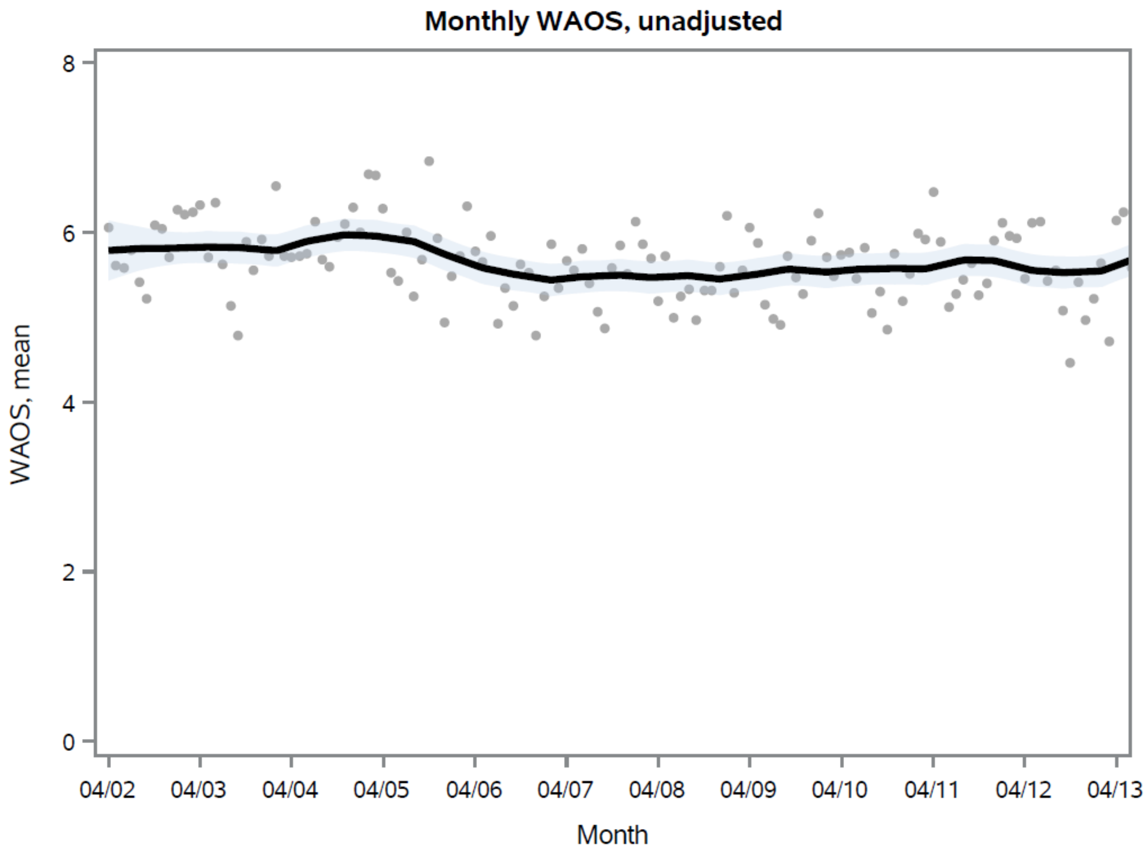

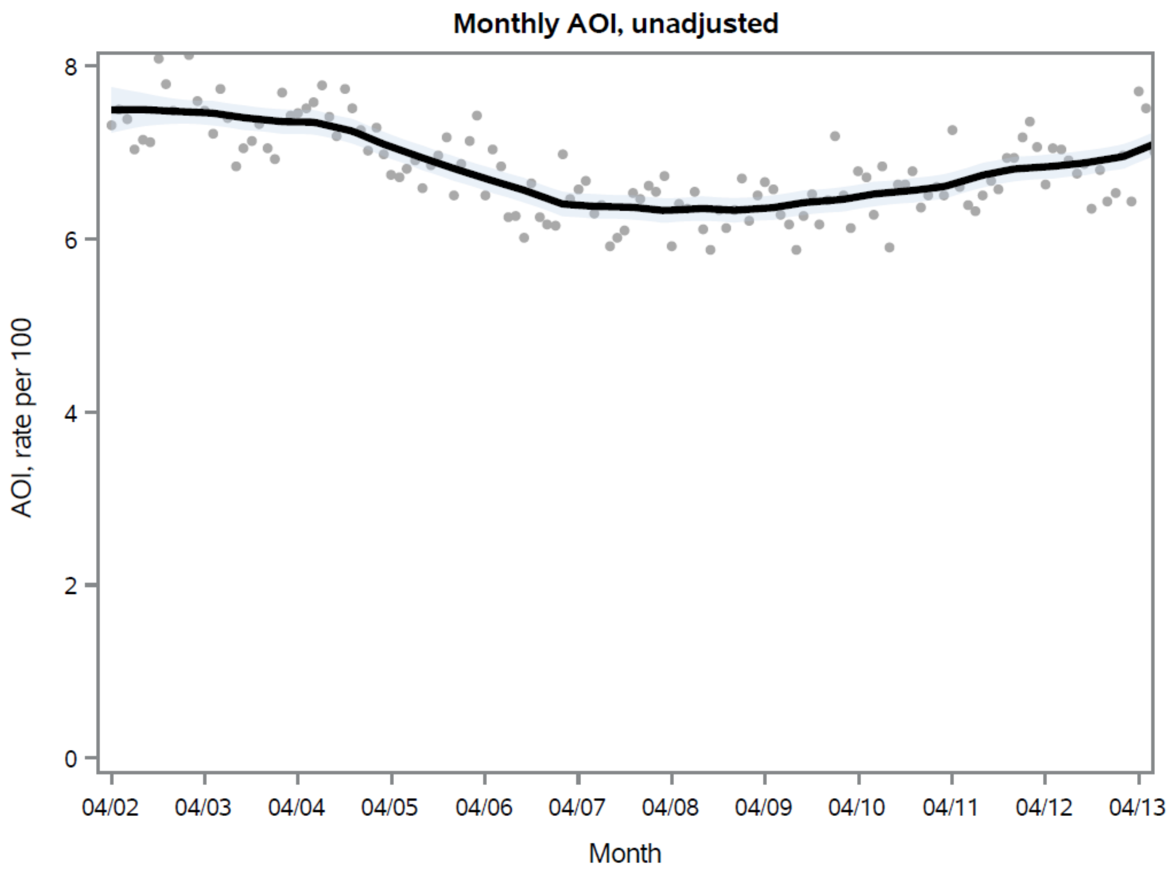

Monthly AOI, unadjusted
AOI, rate per 100
0
2
4
6
8
04/02
04/03
04/04
04/05
04/06
04/07
04/08
04/09
04/10
04/11
04/12
04/13
Month

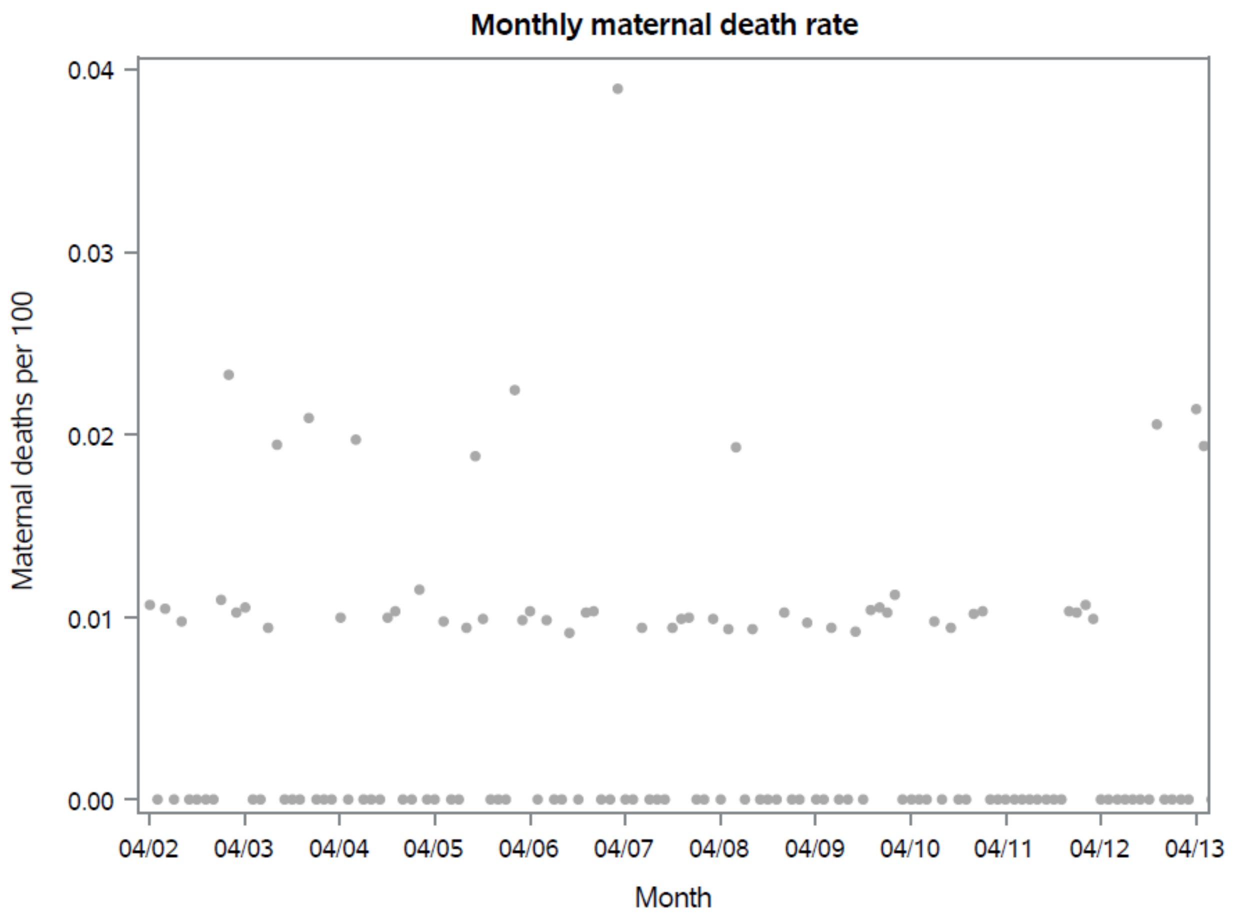
Monthly maternal death rate
Maternal deaths per 100
0.04
0.03
0.02
0.01
0.00
04/02
04/03
04/04
04/05
04/06
04/07
04/08
04/09
04/10
04/11
04/12
04/13
Month

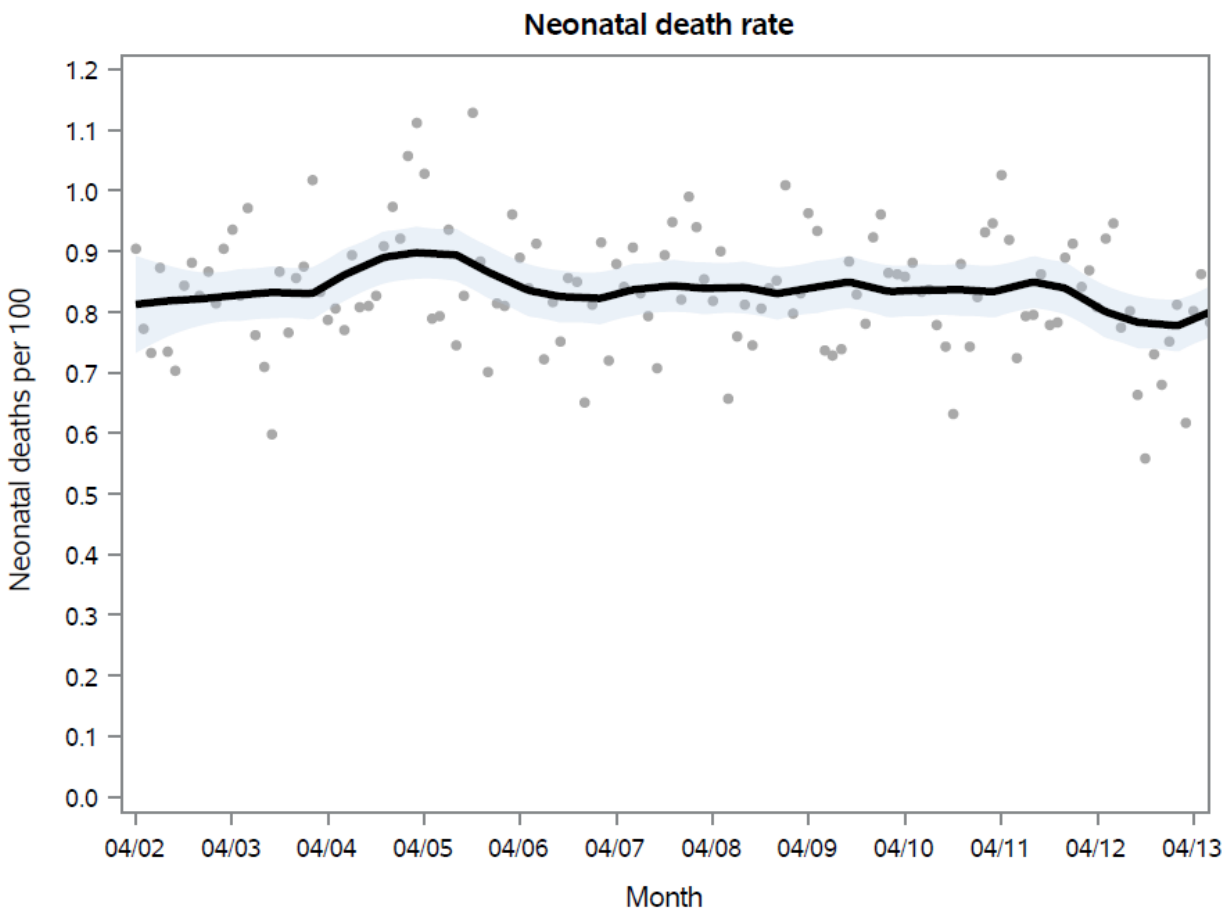
Neonatal death rate
Neonatal deaths per 100
1.2
1.1
1.0
0.9
0.8
0.7
0.6
0.5
0.4
0.3
0.2
0.1
0.0
04/02
04/03
04/04
04/05
04/06
04/07
04/08
04/09
04/10
04/11
04/12
04/13
Month

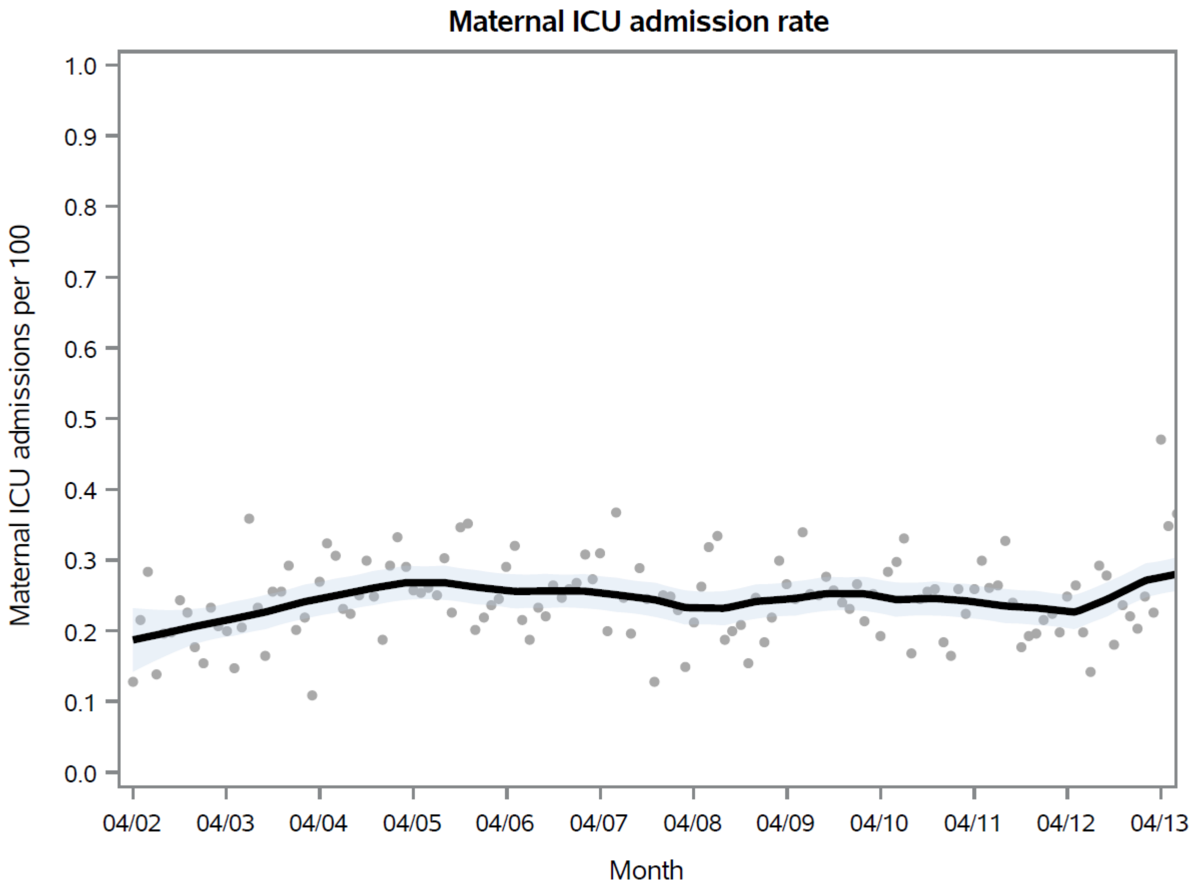

Maternal ICU admission rate
Maternal ICU admissions per 100
1.0
0.9
0.8
0.7
0.6
0.5
0.4
0.3
0.2
0.1
0.0
04/02
04/03
04/04
04/05
04/06
04/07
04/08
04/09
04/10
04/11
04/12
04/13
Month

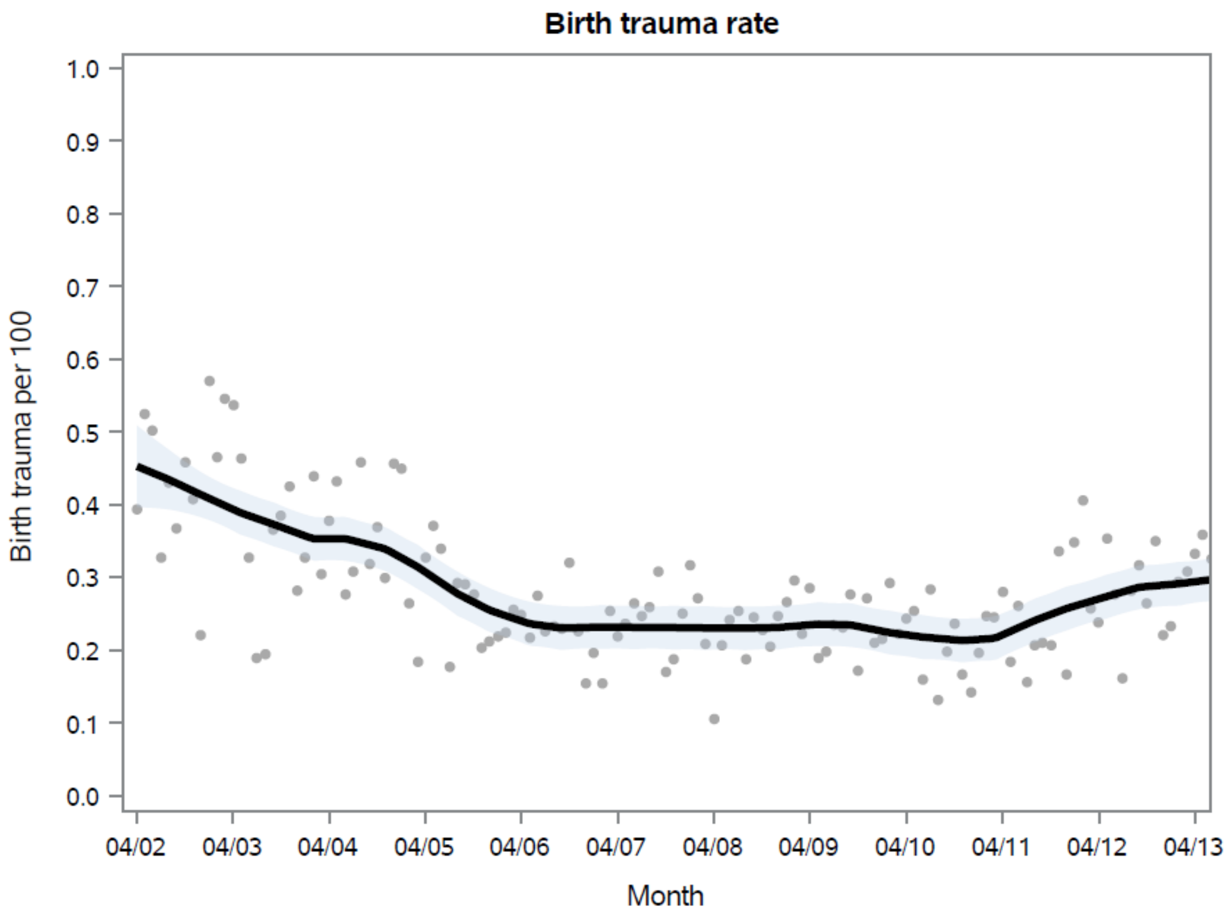

Footnote: Neonatal birth trauma includes cerebral hemorrhage due to birth injury, skeletal injuries due to birth, injuries to spine and spinal cord, brachial plexus, facial nerve or other nerves.

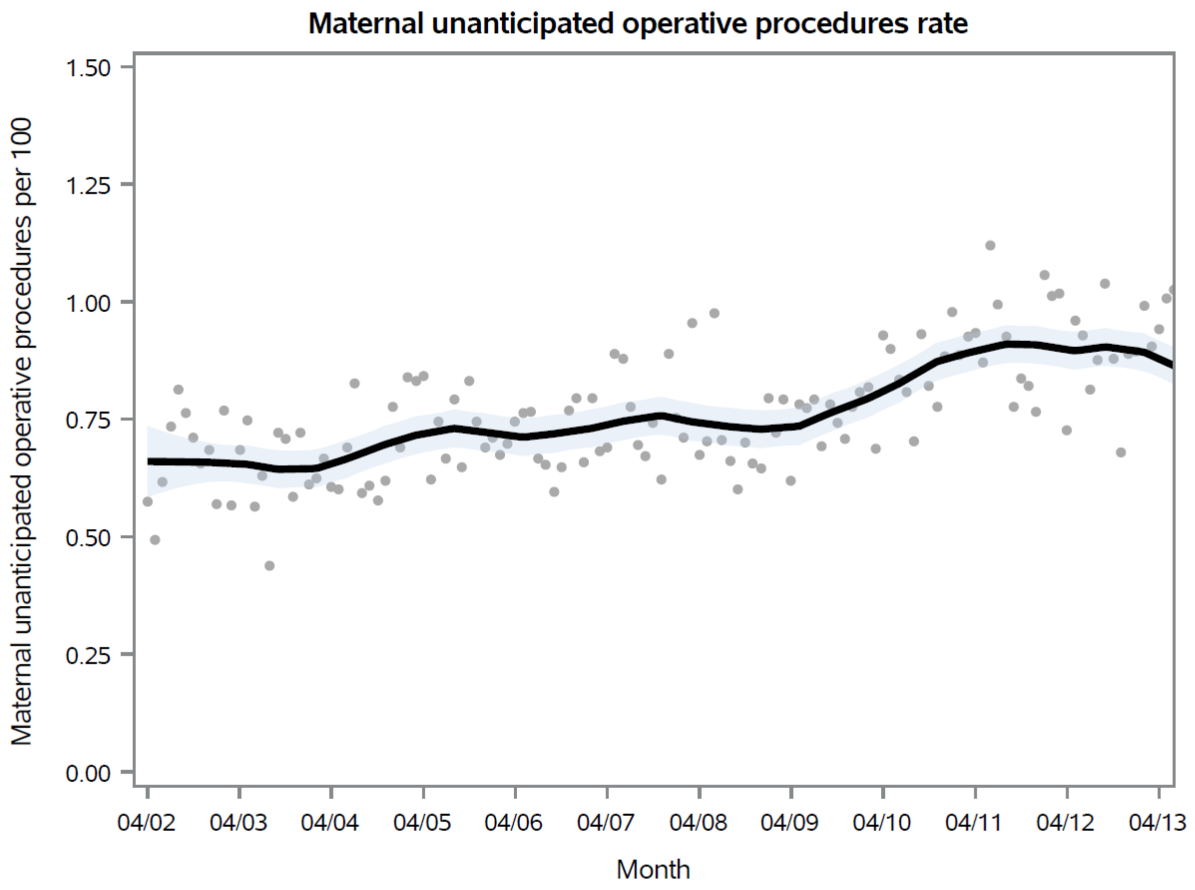

Footnote: Includes procedures to control post-partum bleeding such as D and C following delivery, control of post-partum hemorrhage by ligation or embolization, hysterectomy, etc.

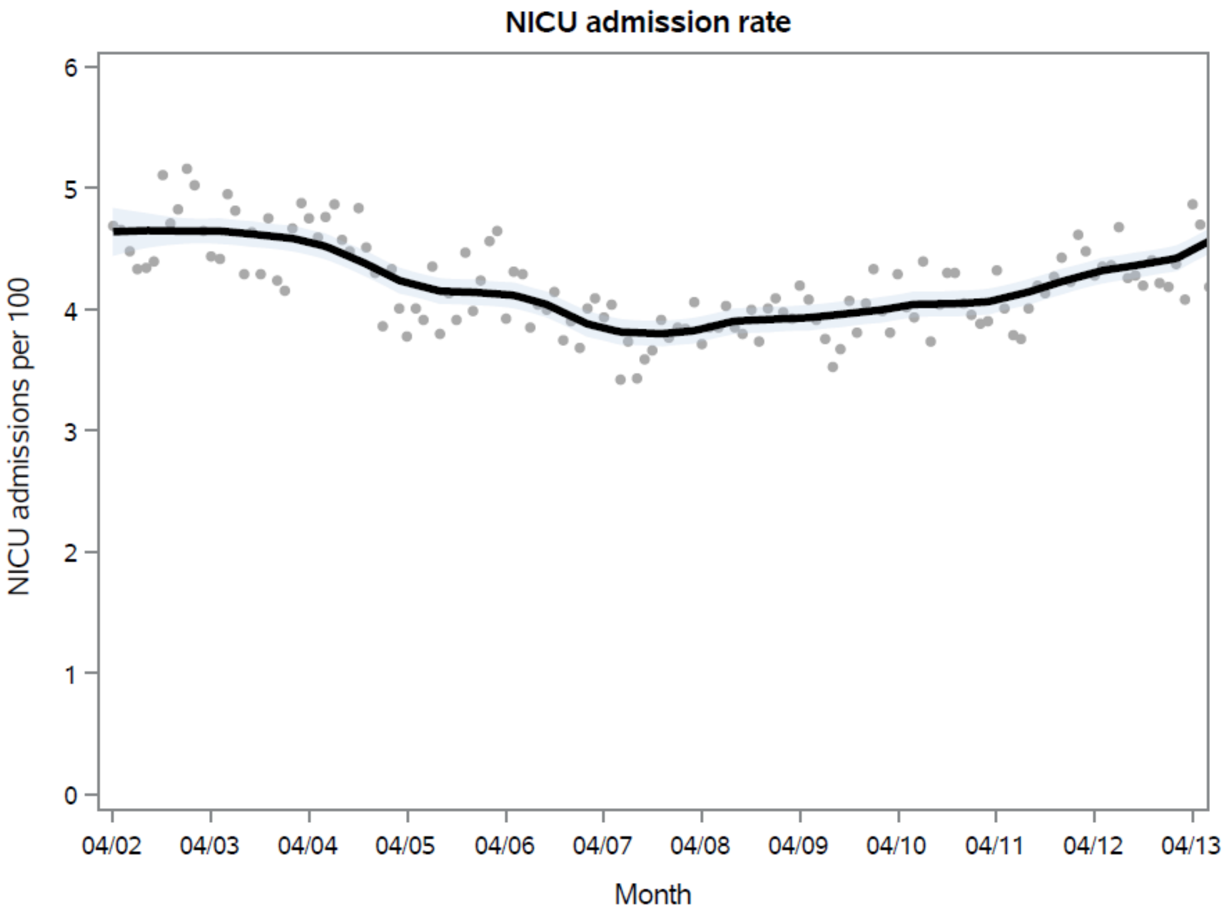
NICU admission rate
NICU admissions per 100
0
1
2
3
4
5
6
04/02
04/03
04/04
04/05
04/06
04/07
04/08
04/09
04/10
04/11
04/12
04/13
Month

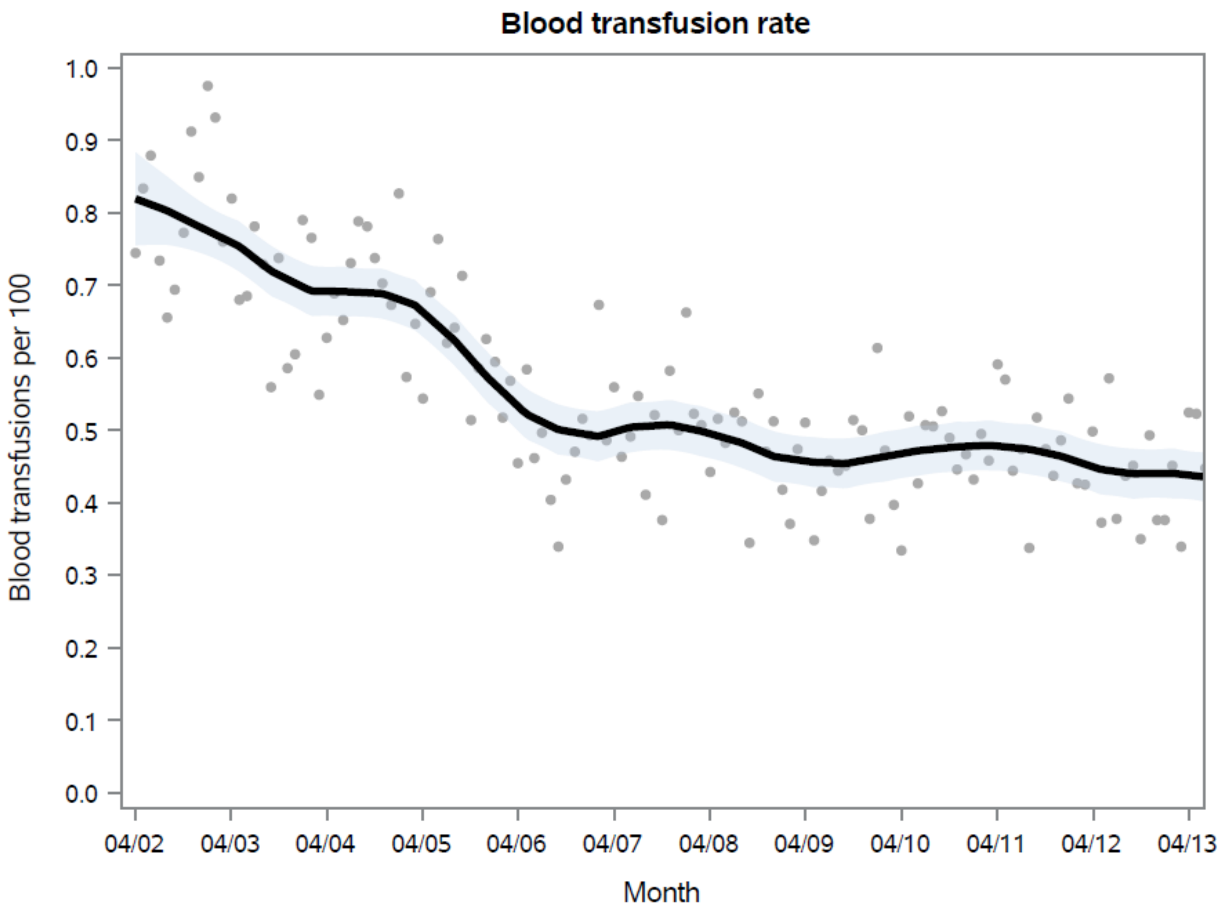
Blood transfusion rate
Blood transfusions per 100
1.0
0.9
0.8
0.7
0.6
0.5
0.4
0.3
0.2
0.1
0.0
04/02
04/03
04/04
04/05
04/06
04/07
04/08
04/09
04/10
04/11
04/12
04/13
Month

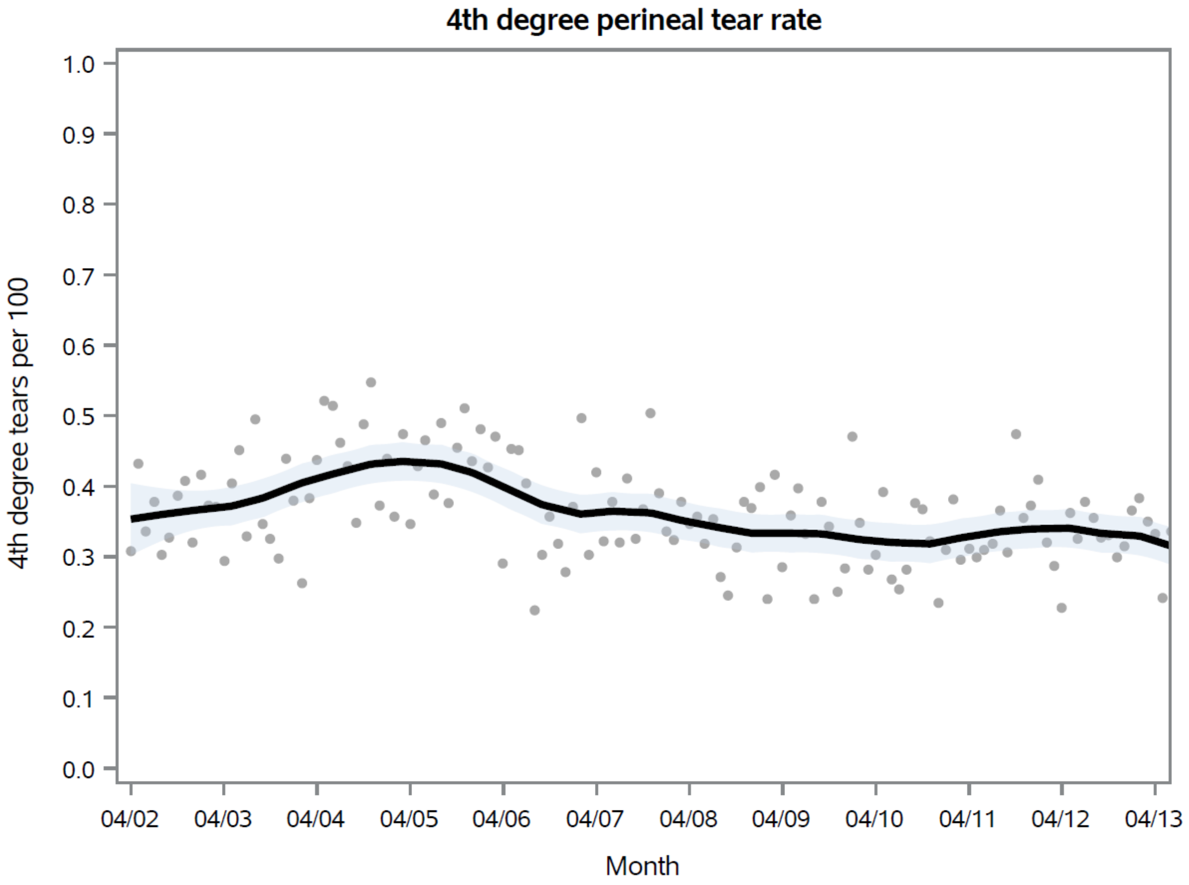
4th degree perineal tear rate
4th degree tears per 100
1.0
0.9
0.8
0.7
0.6
0.5
0.4
0.3
0.2
0.1
0.0
04/02
04/03
04/04
04/05
04/06
04/07
04/08
04/09
04/10
04/11
04/12
04/13
Month
