# Supplementary material for: Effect of implementation of the MOREOB program on adverse maternal and neonatal birth outcomes in Ontario, Canada: a retrospective cohort study
Source: BMC Pregnancy Childbirth. 2019 May 3;19:151. doi: 10.1186/s12884-019-2296-5 (PMC6500060; doi:10.1186/s12884-019-2296-5)
Supplement: Supplementary file 3 — Characteristics of the hospitals included in the analysis, and the characteristics for the full sample of hospitals enrolled in the MOREOB program. (DOCX 14 kb) [file 12884_2019_2296_MOESM3_ESM.docx]

#### Additional file 3. Characteristics of the hospitals included in the analysis, and the characteristics for the full sample of hospitals enrolled in the MORE^OB^ program.

| Level of care, n (%) | | Included hospitals, n=55 | Full sample of hospitals, n=63 |
| --- | --- | --- | --- |
|  | 1 | 12 (21.8) | 20 (31.7) |
|  | 2 | 37 (67.3) | 37 (58.7) |
|  | 3 | 6 (10.9) | 6 (9.5) |
| Birth Volume, n (%) | |  |  |
|  | ≤ 250 | N/A | 8 (12.7) |
|  | 251-500 | 6 (10.9) | 6 (9.5) |
|  | 501-1000 | 10 (18.2) | 10 (15.9) |
|  | 1001-2499 | 17 (30.9) | 17 (27.0) |
|  | 2500-4000 | 15 (27.3) | 15 (23.8) |
|  | >4000 | 7 (12.7) | 7 (11.1) |
