# Supplementary material for: Effect of implementation of the MOREOB program on adverse maternal and neonatal birth outcomes in Ontario, Canada: a retrospective cohort study
Source: BMC Pregnancy Childbirth. 2019 May 3;19:151. doi: 10.1186/s12884-019-2296-5 (PMC6500060; doi:10.1186/s12884-019-2296-5)

Additional file 4: Underlying secular trend for WAOS and mAOI, using time modeled as a continuous with a restricted cubic spline function

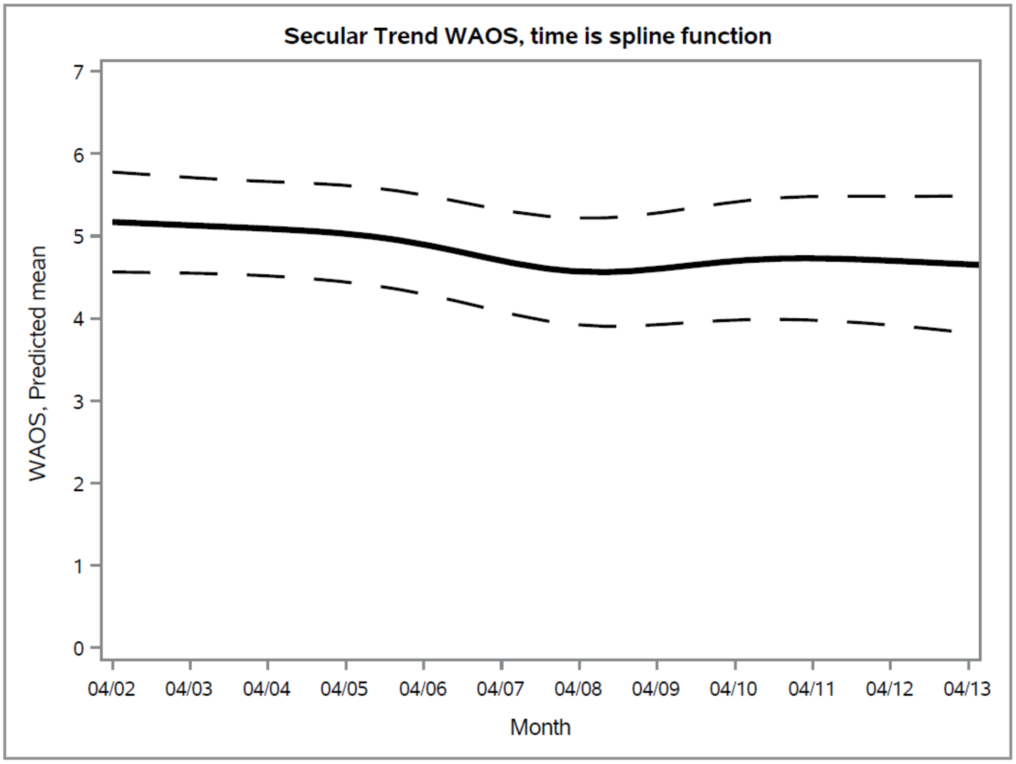

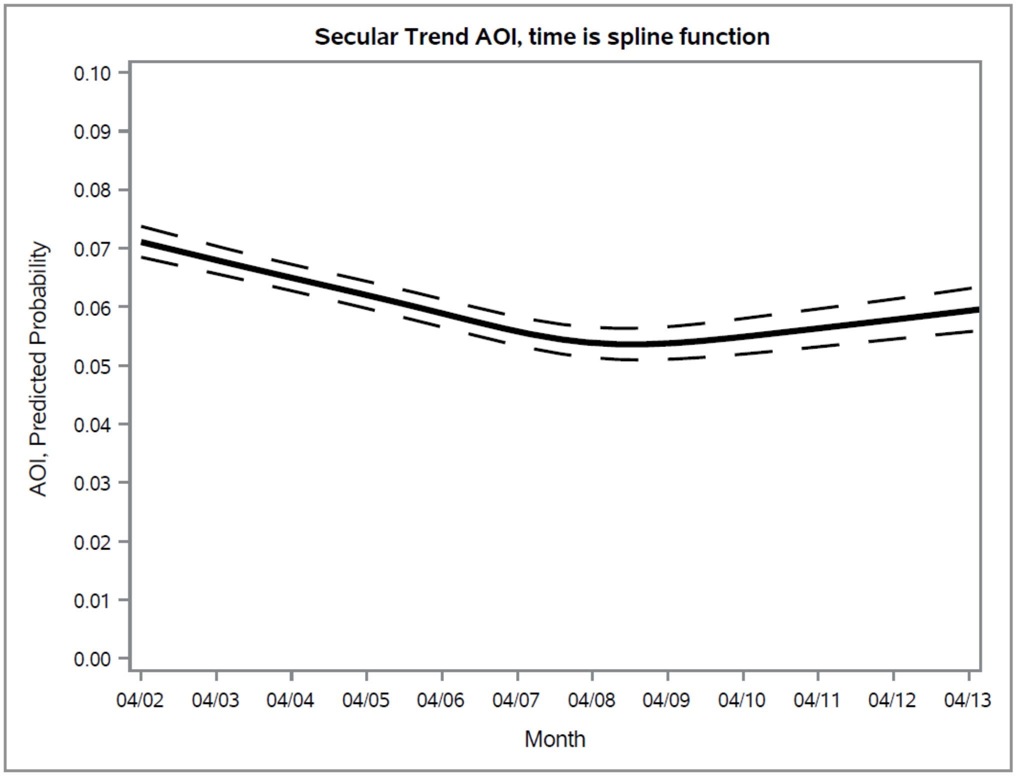

Supplement: Supplementary file 4 — Underlying secular trend for WAOS and mAOI, using time modeled as a continuous with a restricted cubic spline function. (PDF 214 kb) [file 12884_2019_2296_MOESM4_ESM.pdf]
