# Supplementary material for: Effect of implementation of the MOREOB program on adverse maternal and neonatal birth outcomes in Ontario, Canada: a retrospective cohort study
Source: BMC Pregnancy Childbirth. 2019 May 3;19:151. doi: 10.1186/s12884-019-2296-5 (PMC6500060; doi:10.1186/s12884-019-2296-5)
Supplement: Supplementary file 5 — Results of sensitivity analyses for association between MOREOB implementation and two primary study outcomes. This table shows the Results of sensitivity for association between MOREOB implementation and two primary study outcomes. (DOCX 14 kb) [file 12884_2019_2296_MOESM5_ESM.docx]

**Additional file 5: Results of sensitivity for association between MORE^OB^ implementation and two primary study outcomes**

| **Outcome** | **Sensitivity model^*^** | |
| --- | --- | --- |
|  | **Does not account for between-hospital heterogeneity** | **Accounts for between-hospital heterogeneity** |
| **mAOI, OR (95% CI)** | 1.17 (1.10 to 1.25)^**^ | 1.13 (0.96 to 1.33) |
| **WAOS, mean change (95% CI)** | 0.50 (0.02 to 0.99)^**^ | 0.36 (-0.25 to 0.97) |

**^*^**Sensitivity analysis: time is modeled as a categorical variable rather than continuously, using restricted cubic spline, as was done in the main analysis.

^**^p<0.05
